# HIV-Infected Patients: Cross Site-Specific Hydrolysis of H3 and H4 Histones and Myelin Basic Protein with Antibodies against These Three Proteins

**DOI:** 10.3390/molecules26020316

**Published:** 2021-01-09

**Authors:** Svetlana V. Baranova, Pavel S. Dmitrenok, Valentina N. Buneva, Sergey E. Sedykh, Georgy A. Nevinsky

**Affiliations:** 1 Institute of Chemical Biology and Fundamental Medicine, Siberian Division of Russian Academy of Sciences, Lavrentiev Ave. 8, 630090 Novosibirsk, Russia; swb@ngs.ru (S.V.B.); buneva@niboch.nsc.ru (V.N.B.); sedyh@niboch.nsc.ru (S.E.S.); 2 G. B. Elyakov Pacific Institute of Bioorganic Chemistry, Far Eastern Brunch of the Russian Academy of Sciences, 159 Pr. 100 let Vladivostoku, 690022 Vladivostok, Russia; paveldmt@piboc.dvo.ru

**Keywords:** HIV infected patients, human blood antibodies, catalytic antibodies, hydrolysis of H3, H4 histones, and myelin basic protein, cross-complexation and catalytic cross-reactivity

## Abstract

Histones play important roles in chromatin functioning and gene transcription, but in the intercellular space, they are harmful since they stimulate systemic inflammatory and toxic responses. Electrophoretically homogeneous IgGs against myelin basic protein (MBP), as well as H3 and H4 histones, were isolated from sera of HIV-infected patients. In contrast to known classical proteases, these IgGs split exclusively only histones and MBP but no other control proteins. Among 13 sites of hydrolysis of H3 by IgGs against H3 and 14 sites for anti-MBP IgGs, only two sites of the hydrolysis were the same. Between seven cleavage sites of H4 with IgGs against H4 and 9 sites of this histone hydrolysis by antibodies against MBP, only three sites were the same. The sites of hydrolysis of H3 (and H4) with abzymes against these histones and against MBP were different, but several expended protein clusters containing hydrolysis sites are partially overlapped. The existence of enzymatic cross-reactivity of abzymes against H3 and H4 and MBP represents a great menace to humans since due to cell apoptosis, histones constantly occur in human blood. They can hydrolyze MBP of the myelin sheath of axons and play a negative role in the pathogenesis of HIV-infected patients.

## 1. Introduction

Histones and their post-translational modifications possess a crucial role in the functioning of chromatin; it is the remodeling and transcription of genes. Besides, extracellular free histones act as damage molecules [1]. Administration of exogenous histones to animals leads to systemic toxic responses through inflammatory pathways and Toll-like receptors activation [1]. Treatment of mice with anti-histone thrombomodulin, neutralizing antibodies (Abs), activated protein C, and heparin results in the protection of animals against sepsis, lethal endotoxemia, trauma, ischemia-reperfusion injury, peritonitis, pancreatitis, stroke, coagulation, and thrombosis. Moreover, an increased level of nucleosome fragments and free histones in blood sera is connected with several pathophysiological processes, including progression in cancer, and several autoimmune and inflammatory diseases [1].

A central tetramer consisting of two both molecules of H3 and H4 histones in the core of nucleosome particles is surrounded by two dimers of H2B and H2A histones tied with two double-stranded DNA superhelical turns [2]. The H3 and H4 histones were possessed the maximum transfection efficiency of all tested agents [3]. In addition, it was shown that post-transcriptional modifications of histones are associated with chromatin structure changes leading to regulation of transcription processes. In systemic lupus erythematosus (SLE), anti-DNA autoantibodies (auto-Abs) are directed against DNAs of histone-DNAs nucleosomal complexes appearing in the blood due to cell apoptosis [4].

HIV-1 is the etiologic agent causing a very dangerous disease, human autoimmune deficiency syndrome (AIDS) ([5], and references therein). HIV-1 and some other viruses inject into organisms of humans various foreign DNAs, RNAs, proteins, polysaccharides, lipids, and other components. The developments of autoimmune diseases lead to the production of auto-Abs due to activation of different B-cell and molecular mimicry between microbial or viral and host antigens and specific expression of immunoregulatory molecules, as well as the anti-idiotypic network [6,7,8,9]. Activation of B lymphocytes by HIV-1 components leads to the synthesis of auto-Abs to different components of cells: DNA, RNA, small nuclear ribonucleoproteins, thyroid peroxidase, cardiolipin, thyroglobulin, β_2_–glycoprotein I, myosin and erythropoietin, as well as Abs to several viral proteins including viral integrase (IN), and reverse transcriptase ([5,10] and references therein). In contrast to polylysine, H1, and H2A histones, only H3 and H4 histones are able to promote transfer in Jurkat cells HIV-1 Tat gene; it was supposed that histones H3 and H4 in eukaryotic cells could catalyze the gene transfer [3]. 

Using ELISA, relative concentrations of Abs against five histones (H1, H2A, H2B, H3, and H4) [11] and against MBP [12] in the sera of HIV-infected patients were estimated. The titers of Abs against histones and MBP were shown to be statistically significantly higher in comparison with those in healthy donors. The ability of some Abs molecules to bind non-specifically during ELISA or affinity chromatographies on specific sorbents, a large panel of diverse antigens, is known as complexation polyspecificity or polyreactivity of Abs [13]. In some cases, this may be a consequence of a significant structural similarity of certain molecules, including the homology of sequences of polymeric molecules. Therefore, ELISA data on the relative content of certain components in the analyzed mixtures can be overestimated and refers to the sum of the content of structurally similar components.

Artificial Abs-abzymes (Abzs) against stable analogs of reaction transition states of more than 190 different chemical reactions were described (reviewed in [14,15,16]). Natural Abs splitting oligosaccharides, DNAs, and RNAs, different peptides, and proteins were found in sera of patients with several autoimmune and viral diseases [16,17,18,19,20,21,22,23,24,25,26,27,28,29,30,31,32,33,34,35]. Abzymes with very low activities hydrolyzing thyroglobulin [21], vasoactive neuropeptide [23], and polysaccharides [29] were found in sera of some healthy humans. Nevertheless, healthy humans usually lack abzymes [16,17,18,19,20,21,22,23,24,25,26,27,28,29,30,31,32,33,34,35]. Some germline Abs of healthy humans, however, can possess high levels of amyloid-directed, promiscuous, and superantigen-directed activities and microbe-directed and autoantigen-directed specificities [30,31].

The sera of HIV-infected patients comprise Abs against viral components and auto-Abs to many different human cell components ([5,19] and references therein). Sera of HIV-infected patients contain IgGs and/or IgMs hydrolyzing DNA [36], MBP [12], histones [37,38,39], HIV-1 integrase [40,41,42,43], and reverse transcriptase [44]. It is important that 100% of Abs from sera of 32 HIV-infected patients efficiently hydrolyze from one to five human histones (H4, H3, H2A, H2B, and H1) [37,38,39]. Thus, Abzs against five histones and MBP may play on their own a very negative role in the HIV-infected pathogenesis. 

It is believed that the development of different autoimmune diseases may be associated with infection of humans with different bacteria or viruses (human herpesvirus, human endogenous retroviruses, and Epstein-Barr virus), producing different superantigens (for review see [6,7,8,9,10,44,45]). There can at first be a synthesis of Abs against viral or bacterial components that may be structurally resembling human components [45,46]. Then, because of the mimicry of any viral and bacterial proteins with those of human ones, the immune system can result in violation leading to the generation of auto-Abs to human components and the development of autoimmune diseases. 

First, it was shown that immunization of different autoimmune mice leads to a dramatically higher incidence of abzymes production with higher enzymatic activities than in normal, conventionally used mouse strains [47,48]. In addition, it was suggested that this phenomenon of higher incidence of abzymes production and development of AIDS are originated from specific defects of hematopoietic stem cells (HSCs) [49]. Later, it was proven that the spontaneous and antigen-induced development of AIDS in autoimmune prone mice occurs due to immune reorganization of bone marrow HSCs. Defects of the bone marrow include specific changes in the profile of differentiation of bone marrow HSCs, leading to the appearance of B-cells producing abzymes hydrolyzing DNA, polysaccharides, peptides, and proteins [50,51,52,53]. The appearance of auto-Abs with enzymatic activities is the earliest and statistically most significant marker of many AIDS in humans and mammals [18,19,20,21,50,51,52,53]. The formation of lymphocytes synthesizing abzymes occurs already in the cerebrospinal fluid of patients with AIDS. Moreover, the relative activities of abzymes obtained from the cerebrospinal fluid of MS patients, hydrolyzing MBP, DNA, and oligosaccharides depending on their substrate, is about 40–60 times higher than Abs found in the blood of the same patients [54,55,56].

Production of auto-Abs with cross-catalytic reactivity between different viral and human proteins, as well as various human proteins, could be very dangerous for the development of many AI diseases, including HIV-infected patients. Non-specific complexation of the unspecific and sequence-specific enzymes with foreign substrates and ligands is a widespread phenomenon [57,58,59,60,61,62]. The efficiency of the selection of the correct substrates by enzymes due to the stage of complexation is only 1–2 and very rarely three orders of magnitude [57,58,59,60,61,62]. It is subsequent stages of changes in substrate conformation, and directly the stage of the catalysis provides an increase in the rate of the reaction by 5–8 orders of magnitude in the case of specific in comparison with the non-specific substrates [57,58,59,60,61,62]. One of the extremely important factors determining the increase in the reaction rate under the action of enzymes is “orbital control-fitting” of the reacting groups of the enzyme and substrate with an accuracy of 10–15°, which is possible only for specific substrates. Therefore, in contrast to non-specific complexation, which is also found in some canonical enzymes, catalytic cross-reactivity is an extremely rare case [57,58,59,60,61,62]. Typically, classical enzymes catalyze only one chemical reaction.

In many articles, it was shown that abzymes against many proteins usually hydrolyze only one specific protein-antigen and cannot split many other control globular proteins ([18,19,20,21] and references therein). It was shown that histones- and MBP-hydrolyzing activities are the own properties of IgGs from the blood sera of HIV-infected [11,12,37,38,39]. According to previous data, anti-MBP auto-Abs from sera of patients with several autoimmune diseases can hydrolyze only MBP [26,27,28,32,33,34,35], while anti-histones abzymes can split only histones [11,12,37,38,39]. 

Still, there has been no accumulated data on catalytic cross-activity of any abzymes against different proteins [16,17,18,19,20,21]. But recently, it was first shown that HIV-infected IgGs against MBP hydrolyze, not only MBP, but also H1 (and vice versa) abzymes against H1 effectively hydrolyze MBP [12]. Similar results concerning complexation polyreactivity and cross-hydrolysis were obtained for antibodies against MBP and IgGs against H2A and H2B antibodies from the blood of HIV-infected patients [63]. Interestingly, the ELISA data of anti-MBP antibody preparations demonstrated seemingly non-zero content of anti-H1, anti-H2A, and anti-H2B antibodies, and vice versa anti-H1, anti-H2A, and anti-H2B gave a low but positive response to anti-MBP antibodies [12,63]. It turned out that protein sequences of H1, as well as H2A, and H2B histones are characterized by an increased level of their homology MBP [12,63]. The main indicator of the absence in anti-MBP of anti-H1 IgGs (as well as anti-H2A and H2B) was that they hydrolyzed these substrates, but at different specific sites [12,63].

We could not exclude that enzymatic cross-reactivity may be not only for auto-Abs against MBP and H1 (as well as H2A and H2B histones), but also against other histones, including H3 and H4. Complexes of histones with DNA appear in the blood of healthy and sick humans constantly due to the apoptosis of various cells. When cross-enzymatic activity between MBP and histones exists, Abzs against histones can hydrolyze the myelin basic protein of axonal envelopes of nerve tissues. This may be an inside factor of humans in the development of contravention of the nervous system in patients with various autoimmune pathologies, including HIV-infected patients.

In this study, we analyzed possible enzymatic cross-reactivity IgGs against histones H3 and H4 histones in the splitting hydrolysis of MBP, while Abs against MBP in the hydrolysis of H3 and H4 histones. Interestingly, IgGs against H3, H4, and MBP possess no catalytic cross-reactivity with respect to other various control proteins. Specific sites of the hydrolysis of H3, H4 histones by IgGs against these proteins and ant-MBP Abs using MALDI mass spectrometry were determined.

## 2. Results 

### 2.1. Purification of Antibodies

IgGs from sera of HIV-infected patients were first isolated by affinity chromatography of sera proteins on Protein G-Sepharose in special conditions removing non-specifically bound proteins [32,33,34,35]. Then IgG samples were additionally purified using FPLC gel filtration under conditions destroying immune complexes (buffer pH 2.6) as in [32,33,34,35]. It was shown recently that all 32 IgGs preparations of HIV-infected patients efficiently hydrolyze from one to five human histones [11,12]. In this study, to analyze catalytic cross-reactivity of IgGs against MBP, H3, and H4 antibodies in the hydrolysis of MBP and two histones were used electrophoretically homogeneous IgG preparations of HIV-infected patients. 

**Figure 1:**
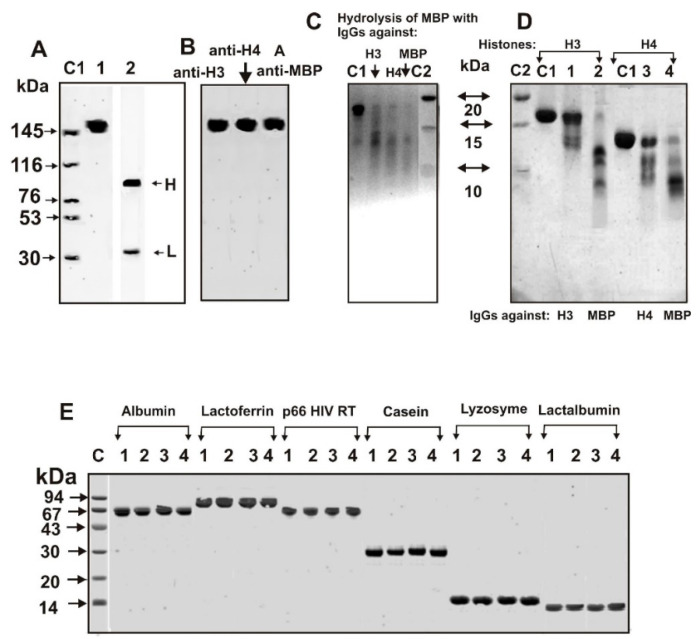
SDS-PAGE analysis of homogeneity of initial IgG_mix_ (12 µg) in 4%–18% gradient gel before (lane 1) and after treatment with dithiothreitol (DTT) (lane 2) (**A**), as well as anti-H3, anti-H4, and anti-MBP Abs after initial IgG_mix_ separation on columns with corresponding affinity sorbents (**B**), followed by silver staining. The arrows (lane C1) indicate the positions of molecular mass markers. SDS-PAGE analysis of MBP hydrolysis with anti-H3 (lane 1) and anti-H4 (lane 2) and anti-MBP IgGs (lane 3) (**C**). SDS-PAGE analysis of H3 hydrolysis with anti-H3 (lane 1) and anti-MBP IgGs (lane 2) as well as H4 hydrolysis with anti-H4 (lane 3) and anti-MBP IgGs (lane 4) (**D**). C1 lanes correspond to MBP (**C**), H3, and H4 (**D**) incubated in the absence of Abs. Lanes C2 correspond to standard proteins with known molecular masses. The hydrolysis of different control proteins in the absence of IgGs (lanes 1) or in the presence of IgGs against H3 (lanes 2), H4 (lanes 3), and MBP (lanes 4) (**E**). Native albumin, human milk lactoferrin, p66 HIV-1 reverse transcriptase (HIV-1 RT), human milk casein, human lysozyme, and human lactalbumin are shown on Panel E. Lane C correspond to proteins with known molecular masses.

The mixture of equimolar amounts of 18 IgGs samples (IgG_mix_) with relatively high activity in the hydrolysis of H3 and H4 histones and MBP was used for separations of auto-Abs preparations against H3, H4, and MBP as described above. Figure 1A,B demonstrate the electrophoretic homogeneity of IgG_mix_ before and after isolation of specific IgGs against H3, H4 histones, and MBP.

Some studies showed the existence of a large number of monoclonal Abs that could bind with a variety of totally unrelated self and foreign antigens (for review, see [13]). However, the affinity of such Abs for the non-specific antigens is usually several orders of magnitude lower than their affinity for the specific antigens. It was shown that all auto-Abs nonspecifically interacting with different affinity sorbents can be eluted from these sorbents by 0.1–0.15 M NaCl [17,18,19,20,21]. Therefore, after affinity chromatography of homogeneous IgGs on histone5H-Sepharose (immobilized all five histones), the fractions eluted with the buffer containing 0.2 M NaCl were not used for further isolation of Abs against H3 and H4 histones. IgGs with higher affinity for this sorbent (a mixture of Abs eluted with 3 NaCl and acidic buffer) were then purified from possible potential impurities of anti-MBP antibodies by these IgGs passing through anti-MBP-Sepharose. IgGs eluted from this sorbent during application were used to obtain specific antibodies against H3 and H4 histones. In this case, after elution of antibodies from H3- and H4-Sepharose, again, only fractions eluted with 3 M NaCl and acid buffer were used for the following analysis.

To obtain antibodies against MBP, affinity chromatography on anti-MBP-Sepharose, the fraction eluted from histone5H-Sepharose upon the application was used. Then, the IgGs fractions were eluted from anti-MBP-Sepharose with 3 NaCl, and the acid buffer was additionally passed through histone5H-Sepharose. For further analysis, we used the fraction that left histone5H-Sepharose upon application (anti-MBP IgGs).

Total IgGs lacking Abs against MBP was applied on H3- or H4-Sepharose; IgGs with a relatively low affinity to these two histones were eluted from the columns with a buffer containing 0.2 M NaCl. Next, all fractions of IgGs with a high affinity for H3 or H4 histones then were eluted with acidic buffer, pH 2.6. It should be mentioned that after described above purification, anti-MBP IgGs were skipped two times using columns containing immobilized H3 and H4 histones, while anti-H3 and anti-H4 IgGs Abs through a column with immobilized MBP. These fractions of anti-MBP, anti-H3, and anti-H4 IgGs were used for the analysis of H3, H4, and MBP hydrolysis.

### 2.2. ELISA of Anti-MBP and Anti-Histones Autoantibodies

The concentration of anti-MBP, as well as anti-H3 and anti-H4 histones IgGs, were measured using homogeneous preparations of anti-MBP, anti-H3, and anti-H4 Abs as in [26,27,28,32,33,34,35]. According to ELISA data, IgGs against MBP gave a positive response (A_450_ units) not only against myelin basic protein (0.28 ± 0.02) but also against H3 (0.08 ± 0.009) and H4 (0.1 ± 0.01). IgG preparations against H3 demonstrated the following data against different proteins (A_450_ units): H3 (0.26 ± 0.05), H4 (~ 0.0), and MBP (0.18 ± 0.04). Antibodies against H4 histones demonstrated (A_450_ units): H4 (0.30 ± 0.03), H3 (~ 0.0), and MBP (0.08 ± 0.009).

Thus, anti-H3 IgG preparations did not contain reliably detectable amounts of Abs against histone H4 and vice versa. However, IgG preparations against MBP showed a reliably detectable positive response against H3 and H4 histones, while anti-H3 and anti-H4 antibodies against MBP. 

This data could be interpreted in two different ways. First, it cannot be excluded that Abs against MBP could not be completely removed from IgGs against H3 and H4 and vice versa. However, the non-specific complex formation of some proteins with antibodies against other ones revealed by ELISA or affinity chromatographies is a widely distributed Abs complexation polyspecificity or polyreactivity phenomenon [13,64,65,66]. Catalysis of different substrates transformation can occur only after their complexes formations with enzymes or abzymes. Moreover, unspecific cross-complexation was also described for many different enzymes and abzymes [58,59,60,61,62,64]. However, most of the canonic enzymes usually catalyze only one chemical reaction; catalysis by enzymes of unspecific ligands transformations after the complexes formation (catalytic cross-reactivity) is an extremely rare case [58,59,60]. All described abzymes against various globular proteins also slip only their specific proteins [17,18,19,20,21,22,23,24,25,26,27,28,32,33,34,35,37,38,39,40,41,42,43,44]. Therefore, it could be assumed that the unspecific complex formation revealed by ELISA or affinity chromatography could be because of a consequence of cross-complexation due to the antigenic determinants homology of protein sequences of MBP with those of H3 and H4 histones. The ELISA data, therefore, could not give an answer about the possibility of the presence in each IgG preparations of admixtures of Abs to other antigens or implementation of the phenomenon of polyspecificity or polyreactivity. In the event of a presence in anti-MBP IgGs preparations in Abs against H3 or H4 abzymes and vice versa, the hydrolysis of H3 or H4 histones should occur at the same specific sites of these proteins cleavage. The only detection of different H3 and H4 cleavage sites with IgGs against these histones and with anti-MBP IgGs may indicate that they possess not only cross-complexation polyreactivity but also enzymatic cross-reactivity. Thus, it was of interest to analyze a possible enzymatic cross-reactivity between Abs-abzymes against H3 and H4 histones and MBP. 

### 2.3. SDS-PAGE Analysis of Catalytic Cross-Reactivity 

Using several chromatographies, including H3-Sepharose, H4-Sepharose, and MBP-Sepharose of electrophoretically homogeneous total IgG preparations, we obtained fractions with a high affinity to H3 histone (anti-H3 IgGs), (anti-H4 IgGs) and to MBP (anti-MBP IgGs). Electrophoretically homogeneous MBP preparations, unfortunately, are not available. Due to cDNA alternative splicing and partial hydrolysis of MBP in the brain of some humans, protein preparations can contain several related protein forms (21.5, 18.5, 17.5, ≤14.0 kDa) and products of their hydrolysis [26,27,28]. C1 line of Figure 1C shows the human MBP starting preparation heterogeneity containing mainly of 14.5–18.5 kDa protein forms. After 12 h of the incubation of MBP with auto-Abs against H3, H4, and MBP, all its forms >15 kDa decreases very much compared to control (C1), and the formation of smaller proteins and peptides is observed (Figure 1C). This may indicate that not only IgGs against MBP, but also to H3 and H4 are able to hydrolyze MBP.

Figure 1D shows that after the incubation of H3 and H4 histones for 12 h with IgGs against these histones and MBP results in their very efficient hydrolysis. At the same time, auto-Abs against H3, H4, and MBP cannot hydrolyze several other control proteins: human albumin and lactoferrin, HIV-1 reverse transcriptase (HIV RT), casein, lysozyme, and human lactalbumin (Figure 1E). 

However, these data still do not provide absolute evidence of enzymatic cross-reactivity between IgGs against MBP, H3, and H4 histones, since, in spite of the several affinity chromatographies of their purifications, it cannot be ruled out that the preparations obtained nevertheless can contain small impurities of alternative antibodies. The clearest evidence of catalytic cross-catalytic activities may be a significant difference in the specific sites of the hydrolysis of H3 and H4 by antibodies against these histones and MBP.

### 2.4. MALDI Analysis of H3 Histone Hydrolysis 

**Figure 2:**
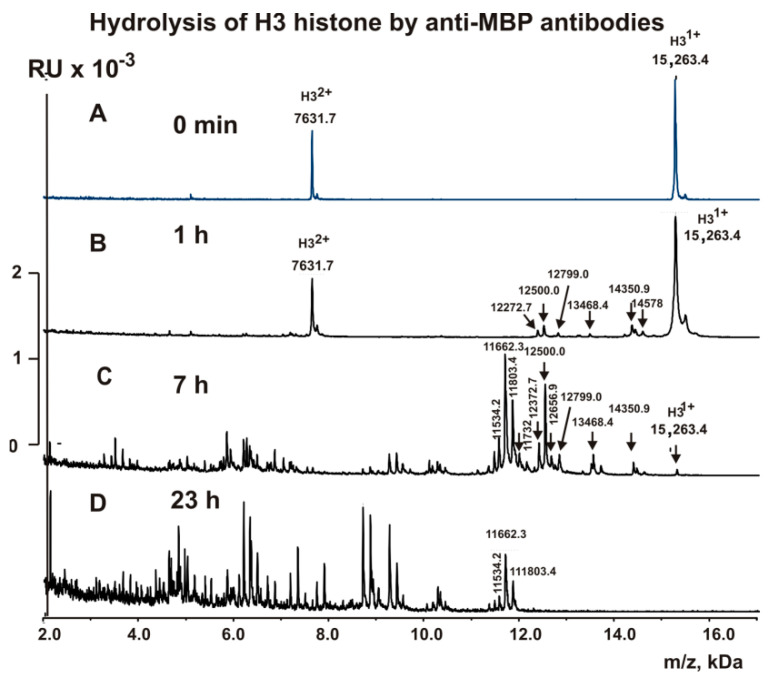
MALDI spectra (**A**–**D**) corresponding to H3 histone (1.0 mg/mL) over time hydrolysis (0–23 h) by anti-MBP IgGs (5.0 µg/mL).

**Figure 3:**
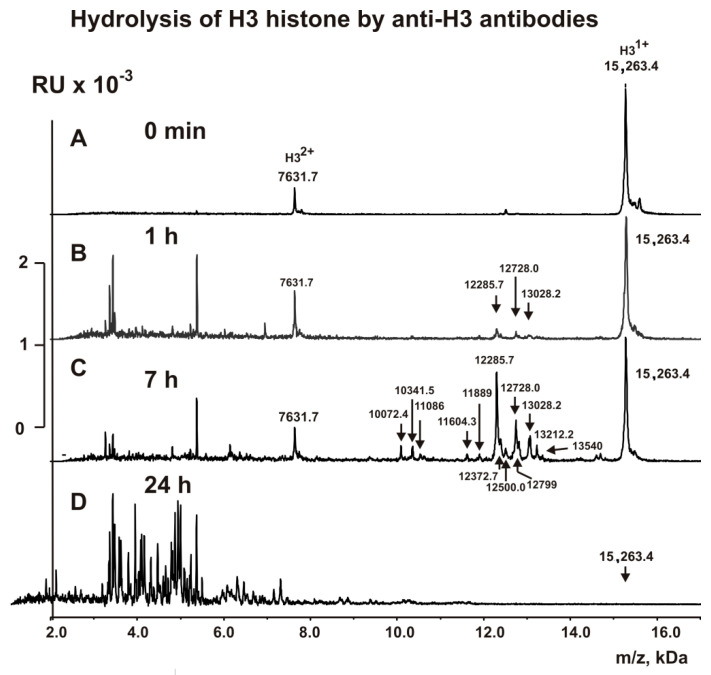
MALDI spectra (**A**–**D**) corresponding to H3 histone (1.0 mg/mL) over time hydrolysis (0–24 h) by anti-H3 IgGs (5 µg/mL)**.**

The fractions with high affinity to H3 histone (anti-H3 IgGs) and to MBP (anti-MBP IgGs) were used to analyze the cleavage sites of H3 histone using MALDI TOFF mass spectrometry. Immediately after the addition of these IgGs (Figure 2 and Figure 3), H3 histone was practically homogeneous and demonstrated two signals of its one- (*m*/*z* = 15,263.4 Da) and of two-charged ions (*m*/*z* = 7631.7 Da). After 1 h of the incubation with anti-MBP IgGs, six very small peaks corresponding to long products of H3 (15,263.4 Da) splitting corresponding to N-terminal part of H3 histone with MMs were revealed (Da): 14,578, 14,350.9, 13,468.4, 12,799.0, 12,500, and 12,272.7 (Figure 2B). The 1 h incubation of H3 with anti-H3 IgGs resulted in only three other small peaks: 13,028.2, 12,728.0, and 12,285.7 Da (Figure 3B). 

Analysis of MMs of all possible sequences H3, which could match these small proteins and peptides, was performed. Finally, we found only one variant of the sequences and cleavage sites of H3 corresponding to every MM of the peptides in the case of anti-MBP IgGs (6T-7A, 8R-9K, 17R-18K, 23K-24A, 26R-27K, 27K-28S) and anti-H3 IgGs (S28-A29, A24-A25, and A21-T22). All these sites of specific hydrolysis of H3 by anti-MBP and anti-H3 IgGs are mostly different. Interestingly, three peaks in the case of anti-H3 IgGs were significantly increased after 7 h of the incubation (Figure 3C), and five statistically significant additional small peaks were revealed (Da): 12,372.7 (K27-S28), 12,500 (R26-K27), 12,799 (K23-A24), 13,212.2 (Q19-L20), and 13,340.3 (K18-Q19). These additional sites of H3 splitting by anti-H3 IgGs should be attributed to the minor ones. 

A somewhat different situation was observed during the hydrolysis of H3 by anti-MBP IgGs. Three peaks (14,578, 14,350.9, and 13,468.4 Da) that appeared at the very beginning of the hydrolysis process did not increase remarkably in the time; they underwent additional hydrolysis at different sites (Figure 2C). On the contrary, three other initial peaks (12,799.0, 12,500, and 12,272.7 Da) to 7 h of the incubation were significantly increased (Figure 2C). All of these cleavage sites correspond to the N-terminal sequence of H3. Interestingly, by 7 h of the incubation, all of these relatively long peptides underwent additional hydrolysis at the C-terminal sequence of H3 at the following sites: C110-A111, A127-R128, R128-R129, I130-R131. After 24 h of incubation, all relatively long peptides underwent additional hydrolysis to short and very short peptides by both anti-H3 IgGs and anti-MBP IgGs (Figure 2 and Figure 3). 

There is no data about possible localization of H3 epitopes; however, according to rough immunoblotting data, the antigenic determinants (AGDs) may possibly be disposed at both the C- and N-terminal terminal regions of H3 [67].

**Figure 4:**
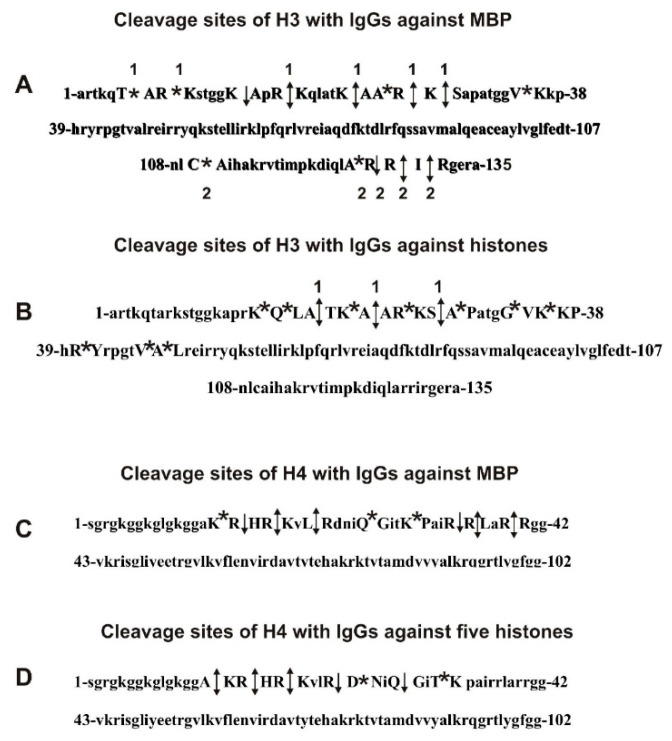
Complete sequence of H3 and H4 histones: all sites of H3 cleavage by anti-MBP (**A**) and anti-H3 histone IgGs (**B**) as well as H4 histone sites of cleavage with anti-MBP (**C**) and anti-H4 histone IgGs (**D**). Major sites of the cleavage are marked shown by double arrows (↕), moderate ones by arrows (↓), and minor sites of the hydrolysis by stars (*) (**A**–**D**).

All sites of H3 splitting by antibodies against H3 histone and MBP are shown in Figure 4A,B. It can be seen that among the sites of hydrolysis of H3 with antibodies against H3 (13 sites) and MBP (14 sites), only two sites coincide (K23-A24 and R26-K27). The site K23-A24 is minor for both antibodies, whereas the R26-K27 site is minor in the case of anti-H3 IgGs but major for anti-MBP IgGs. In addition, anti-MBP IgGs cleaves H3 histone in its N-terminal (four cleavage sites between T6-K18) and C-terminal sequences (four cleavage sites between A127-R131), that is, in sequence areas where there are no histone cleavage sites with anti-H3 IgGs. These data indicate that the anti-MBP IgGs does not contain tangible impurities of anti-H3 IgGs and vice versa. Hydrolysis of substrates can occur only after the formation of a complex of a biocatalyst with its substrate. Consequently, antibodies against H3 histone and MBP possess not only the ability to bind to H3 and MBP but also cross-catalytic activity in the hydrolysis of this histone and MBP.

### 2.5. MALDI Analysis of H4 Histone Hydrolysis

**Figure 5:**
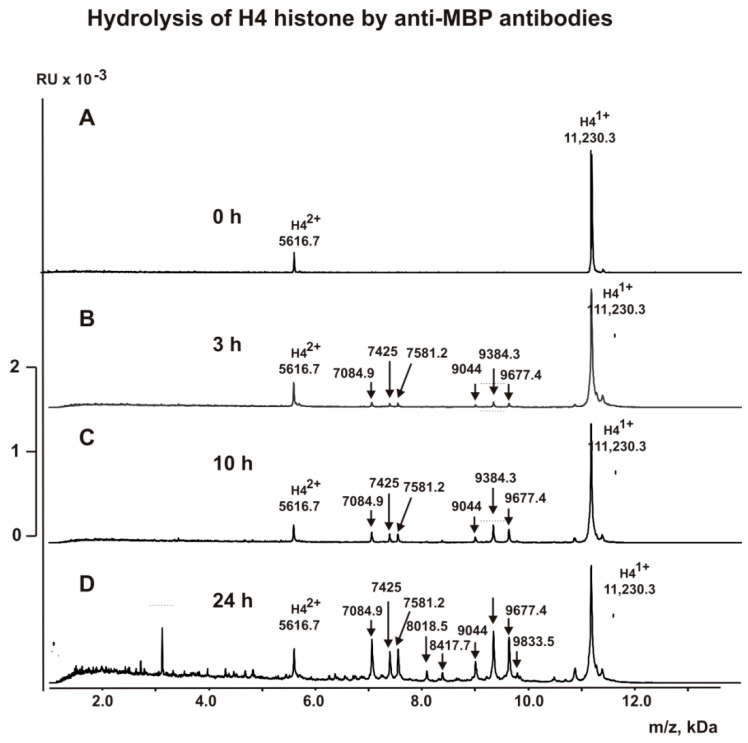
MALDI spectra (**A**–**D**) corresponding to H4 histone (1.0 mg/mL) over time hydrolysis (0–24 h) by anti-MBP IgGs (5.0 µg/mL).

**Figure 6:**
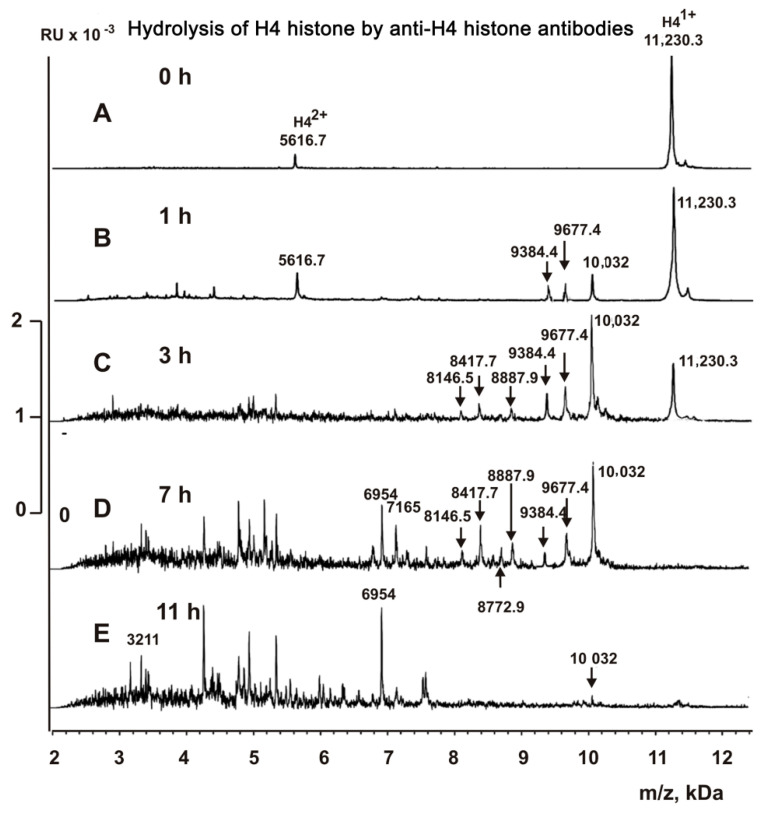
MALDI spectra (**A**–**E**) corresponding to H4 histone (1.0 mg/mL) over time hydrolysis (0–24 h) by anti-H4 IgGs (5 µg/mL).

We have compared all specific cleavage sites of histone H4 by anti-H4 IgGs and anti-MBP IgGs. Before treatment with these IgGs, H4 histone demonstrated two signals of one- (*m*/*z* = 11,230.3) and two-charged ions (*m*/*z* = 5616.7 Da) (Figure 5 and Figure 6). Hydrolysis of H4 by anti-MBP antibodies progressed more slowly than histone H3 (Figure 5). Only after 3 h of the incubation, there was observed six small peaks corresponding oligopeptides of H4 N-terminal part (kDa): 9677.4 (K17-H18), 9384.3 (R19-K20), 9044.0 (L22-R23), 7581.2 (R35-R36), 7425.0 (R36-L37), and 7084.9 (R39-R40) (Figure 5B). After ten hours of incubation, only these six peaks were increased (Figure 5C). And even after 24 h of the incubation, there was an increase mostly only these six peaks, but two new very small peaks appeared: 8417.7 (Q27-G28) and 8018.5 (K31-P32) (Figure 5D). Unlike H3 histone (Figure 4), over time, complete hydrolysis of the starting H4 and its relatively long products of the hydrolysis to a large number of small peptides did not occur (Figure 5D). This indicated the absence in the H4 sequence of a large number of potential sites for its hydrolysis with antibodies against MBP.

IgGs against H4 histone after 1 h of the incubation hydrolyzed of the N-terminal part of H4 histone at three sites (Da): 10,032 (A15-K16) > 9677.4 (R17-H18) ≥ 9384.3 (R19-K20) (Figure 6B). After 3 h of the incubation, these three peaks were significantly increased, and three additional peptides were detected (Da): 8887.9 (R23-D24), 8417.7 (Q27-G28), and 8146.5 (T30-K31) (Figure 6C). However, unlike IgGs against MBP, anti-H4 IgGs almost completely cleaved the initial H4 and intermediate products of its hydrolysis into small peptides during 11 h of incubation (Figure 6E).

All sites of H4 hydrolysis by anti-MBP and anti-H4 IgGs are shown in Figure 4C,D.

Using synthetic oligopeptides, it was shown that one AGD of H4 histone is localized from first to 53 amino acids (AAs), while second in C-terminal part-88–96 AAs of its sequence [68]. Also, it was shown that oligopeptide containing 1–29 AAs of H4 interacts with Abs against H4 histone [69]. 

All thirteen sites of H3 hydrolysis by anti-H3 IgGs can be assigned to one extended cluster: K18-L48 (Figure 4B). Interestingly, to thirteen major, average, and minor sites of H3 hydrolysis by anti-MBP IgGs correspond to two clusters located in the N-(T6-K36) and C-(A127-R131) terminal zones of this protein. The N-terminal zones of these clusters corresponding to the H3 cleavage sites of anti-H3 and anti-MBP IgGs overlap only partially, K18-K36. 

The cluster of seven cleavage sites (A15-K31) of H4 in the case of anti-H4 IgGs (Figure 4) corresponded to an approximately central part of earlier revealed AGD (1–53 AAs) [68] overlaps with a cluster of nine H4 hydrolysis sites for anti-MBP IgGs (K16-R40). However, only three of the sites of hydrolysis of H4 by anti-MBP (9 sites) and by anti-H4 (7 sites) IgGs are the same: R17-H18, R19-K20, and Q27-G28 (Figure 4C,D). Moreover, in the case of IgGs against MBP, there are no major sites of H4 hydrolysis by anti-H4 IgGs (15A-16K and R23-D24), while for anti-H4 IgGs are absent major sites of H4 cleavage by anti-MBP IgGs (R34-R35, R35-L36, and R39-R40). Thus, it is obvious that anti-MBP IgGs do not contain perceptible impurities of anti-H4 IgGs and vice versa. These data indicate that both anti-H4 and anti-MBP can bind to H4 histone and then hydrolyze it showing enzymatic cross-reactivity.

### 2.6. Analysis of Possible Homology of H3 and H4 Histones with MBP 

One of the more possible reasons for the catalytic cross-hydrolysis of H3 and H4 histones and MBP may be a high homology of these histones and MBP. Supplementary Figure S1 shows the identity and similarity of AAs (non-identical Aas, but with highly similar physicochemical properties) of complete sequences of H3 histone and MBP: 24.6% identity, with 47.9% similarity of AAs. The approximately similar situation was observed for H4 histone and MBP sequences: 30.0% identity and 45.0% similarity (Supplementary Figure S1). 

**Figure 7:**
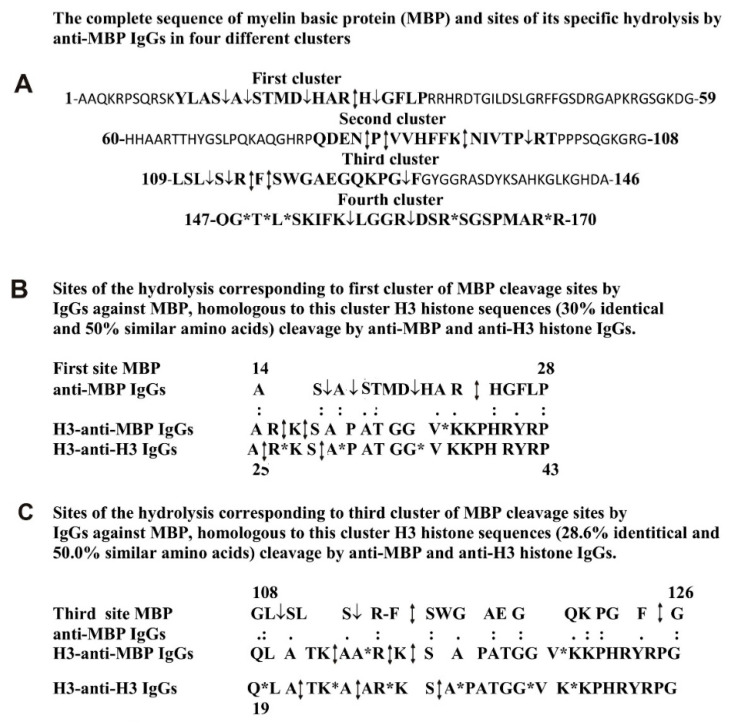
The complete sequence of human MBP and all sites of its hydrolysis in four clusters by anti-MBP IgGs (**A**). Analysis of homology between sequence of the first (**B**) and third (**C**) cleavage sites of MBP corresponding to anti-MBP IgGs with two sequences of H3 histone, which are homologous to these MBP clusters and hydrolyzed by both anti-MBP and anti-H3 histone IgGs. Major sites of the cleavage are marked by double arrows (↕), moderate ones by arrows (↓), and minor sites of the hydrolysis by stars (*) (**A**–**C**).

**Figure 8:**
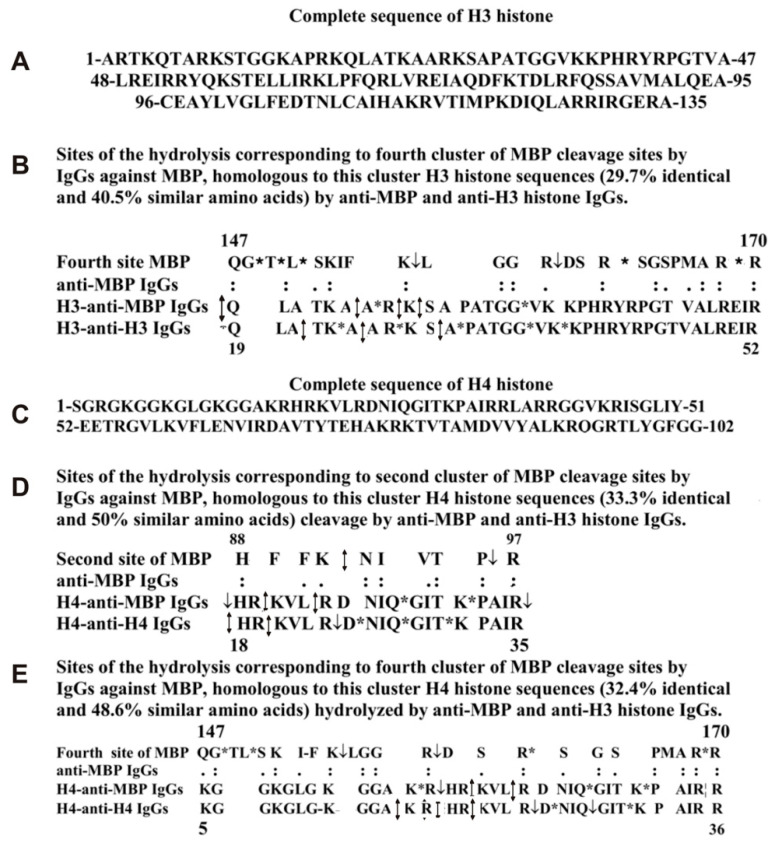
The complete sequence of H3 histone (**A**). Analysis of homology between sequence of the fourth cleavage sites of MBP corresponding to anti-MBP IgGs with one sequence of H3 histone, which are homologous to this MBP cluster and hydrolyzed by both anti-MBP and anti-H3 histone IgGs (**B**). A complete sequence of H4 histone (**C**). Analysis of homology between sequence of the second (**D**) and fourth (**E**) cleavage sites of MBP corresponding to anti-MBP IgGs with two sequences of H4 histone, which are homologous to these MBP clusters and hydrolyzed by both anti-MBP and anti-H4 histone IgGs. Major sites of the cleavage are marked by double arrows (↕), moderate ones by arrows (↓), and minor sites of the hydrolysis by stars (*) (**A**–**C**).

Specific sites for the hydrolysis of myelin basic protein with IgGs against MBP were established previously (Figure 7A) [32,33,34,70]. It turned out that anti-MBP IgGs hydrolyze human MBP at 3–7 of specific sites in four protein clusters corresponding to four antigenic determinants of MBP (Figure 7A). Therefore, it was very interesting to what extent the sequences of these MBP AGD-clusters and their sites of the hydrolysis are homologous with the H3 sequences and the hydrolysis sites of this histone by anti-MBP and anti-H3 IgGs. It turned out that many fragments of the H3 histone sequence have a relatively high level of their homology. Interestingly, several fragments of protein sequences of MBP corresponding to AGDs of MBP showed an increased level of homology with the sequence of histone H3. Three examples of the homology of such sequences are shown in Figure 7B,C and Figure 8B. One can see that MBP sequence corresponding to its first cluster (A14-P28) is homologous to the H3 sequence from A25-P43. It is in this H3 histone A14-P28 fragment there are three sites of histone hydrolysis by Abs against MBP and five sites of H3 splitting by IgGs against H3. 

The third cluster of MBP specific hydrolytic sites (G108-G126) is homogeneous to the Q19-G44 sequence of H3 histone. In these homologous sequences, 4 sites of MBP are hydrolyzed by anti-MBP antibodies; 5 sites of H3 histone hydrolysis correspond to anti-MBP, while 9 sites to anti-H3 abzymes.

The fourth cluster of MBP hydrolysis by anti-MBP IgGs (Q147-R170) demonstrates homology with a Q19-R52 sequence of H3 histone, in which the main part of the sites of H3 hydrolysis with abzymes against this histone is located (Figure 8B). Altogether, all sequences of four different AGDs-clusters of MBP containing 20 sites of its hydrolysis by anti-MBP IgGs show homology with different fragments of the same H3 protein sequence (14A-R30) in which all the histone cleavage sites in the case of anti-H3 antibodies are located. Therefore, Abs against four different AGDs of MBP can bind with varying effectiveness to AGD-sequences of histone H3. This type of alternative binding of anti-H3 antibodies to MBP and anti-MBP abzymes with different fragments of histone sequences may be a reason for the enzymatic cross-polyreactivity of IgGs against MBP and histone H3.

To some extent, a very similar situation occurs in the case of antibodies against H4 histone. It was shown that the first AGD of H4 is localized from 1 to 53 amino acids (AAs), while the second in the C-terminal part-88–96 AAs of its sequence [68]. However, all sites of specific hydrolysis of H4 by IgGs against H4 are located in an A14-P32 fragment of this histone. This A14-P32 sequence of histone H4 demonstrates homology with four sequences corresponding to four AGDs and specific sites of the hydrolysis of MBP by antibodies against MBP. In Figure 8, two typical examples of the homology of the H4 sequences, which are hydrolyzed by antibodies against MBP and against H4, are shown. The first cluster of four specific sites of MBP hydrolysis (H88-R97) by anti-MBP IgGs is homologous to a cluster of three sites of H4 splitting by IgGs against MBP and five sites of its cleavage by anti-H4 IgGs in the fragment H18-R35 (Figure 8D). Figure 8E demonstrates the homology of another K5-R36 fragment of the H4 with MBP sequence Q147-R170 splitting by anti-MBP (four sites); this fragment of H4 contains seven sites of its hydrolysis by Abs against this histone. Anti-MBP abzymes hydrolyze this cluster of H4 histone sequence at six specific sites (Figure 8E). Thus, as in the case of H3, the fragment of H4 histone containing specific hydrolysis sites exhibit homology with four MBP cluster sequences susceptible to hydrolysis of this protein by IgGs against MBP. The H3 and H4 histones clusters of sequences in which the sites their hydrolysis by antibodies against these histones and against MBP are nearly the same, but specific sites of the cleavage are different. 

### 2.7. Affinity of IgGs for Histones

**Figure 9:**
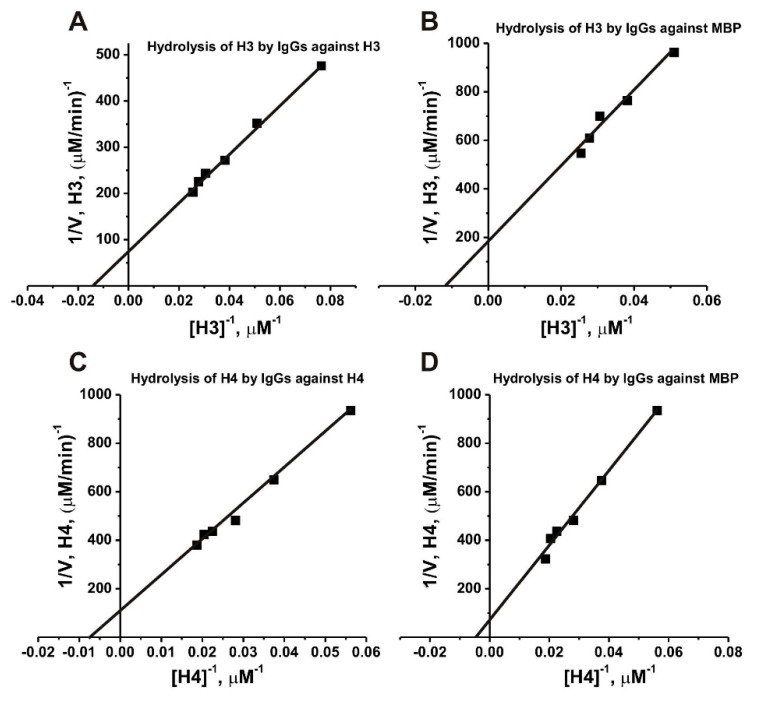
Determination of the *values of K*_m_ and *V*_max_ (*k*_cat_) for H3 and H4 substrates in their hydrolysis by IgGs against MBP (**A**) and (**B**), respectively; 0.04 mg/mL) and by abzymes against H3 (**C**); 0.022 mg/mL) and H4 (**D**; 0.02 mg/mL) using a Lineweaver-Burk plot. The average error in the initial rate estimation at each substrate concentration from two independent experiments did not exceed 10%–20%.

We have estimated the values of *K_m_* and *k_cat_* in the hydrolysis of H3 and H4 by anti-H3, anti-H4, and anti-MBP IgGs (Figure 9). The initial rate data obtained using increasing concentrations of H3 and H4 were consistent with the Michaelis-Menten kinetics. The relative affinities of H3 histone in terms of *K*_m_ values for anti-MBP antibodies (90.0 ± 8.0 µM) was 1.6-fold higher than that for H4 histone (143.0 ± 10.0 µM), while hydrolysis of H4 (*k*_cat_ = 0.037 ± 0.004 min^−1^) was 1.9-fold faster than H3 (*k*_cat_ = 0.02 ± 0.002 min^−1^) (Table 1). The affinity of H3 for anti-H3 IgGs (*K*_M_ = 76.9 ± 8.0 µM) was by a factor of 1.5 higher than that of H4 to anti-H4 IgGs (*K*_M_ = 113 ± 12.2 µM) when *k*_cat_ values were to some extent comparable: 0.09 ± 0.008 and 0.076 ± 0.008 min^−1^, respectively.

## 3. Discussion

The non-specific complexation of some proteins and nucleic acids with Abs against other similar ligands (and some enzymes with various compounds) at ELISA and affinity chromatographies is a widely distributed phenomenon and is known as polyreactivity or polyspecificity of Abs [13,64,65,66]. The difference in the efficiency of binding of specific and non-specific ligands by enzymes and antibodies usually does not exceed one or two orders of magnitude [58,59,60,61,62]. However, canonic enzymes usually catalyze usually only one chemical reaction, and catalysis of enzymes of non-specific ligands (catalytic cross-reactivity) forming complexes with them is an extremely unusual and rare case [58,59,60,61,62]. All described to data catalytic antibodies against various proteins also hydrolyze only their specific globular proteins [16,17,18,19,20,21,22,23,24,25,26,27,28,32,33,34,35,37,38,39,40,41,42,43,44]. Since H3 and H4 histones exhibit homology with MBP, it was possible to suggest that antibodies against these proteins may show cross-complexation during ELISA and affinity chromatography. Obviously, the hydrolysis of any substrates by enzymes or abzymes can be only after their binding to different ligands. However, unspecific cross-complexation with erroneous ligands should not lead to the catalysis of its transformation. Therefore, it was difficult to imagine that IgGs against H3 and H4 histones and MBP can possess not only cross-complexation due to the homology of their protein sequences but also to catalytic cross-reactivity. The first example of enzymatic cross-reactivity was anti-MBP and anti-H1 histone IgGs of HIV-infected patients [12]. Then, catalytic cross-reactivity was shown between anti-MBP abzymes and antibodies against H2A and H2B [63].

Therefore, it was interesting to analyze a possible enzymatic cross-reactivity between Abs-abzymes against H3 and H4 histones and MBP. Using several affinity chromatographies, anti-H3, anti-H4, and anti-MBP antibodies were obtained. As was mentioned above, the non-specific antigens usually have 1–2 orders of magnitude lower affinity than the specific ones. Abs interacting with different various affinity sorbents due to the cross-complexation can be eluted by 0.1–0.15 M NaCl [17,18,19,20,21]. Therefore, after chromatographies, IgGs on different affinity sorbents, the fractions eluted with the buffer containing 0.2 M NaCl were not used for their further purification and analysis. However, this is not enough to be absolutely sure about the absence of impurities in analyzed IgG preparation of Abs to another antigen.

The ELISA data testified in favor of a possibility of the presence in preparations of anti-MBP antibodies IgGs against H3 and H4 and vice versa. However, this data could be a consequence of two different reasons. First, the preparations of anti-MBP antibodies could contain admixtures of IgGs against H3 and H4 and vice versa. Second, each of three preparations of antibodies may not contain noticeable impurities of other ones, but homology between MBP and these histones scilicet their polyspecificity or polyreactivity.

Thus, the ELISA data cannot give an answer about the possibility of the presence in each preparation of IgG impurities to other antigens or realization of the phenomenon of polyspecificity or polyreactivity. However, in the presence of anti-MBP Abs preparations of IgGs against H3 or H4, or in anti-H3 or anti-H4 Abs abzymes against MBP, hydrolysis of H3 and H4 histones should be observed on the specific same sites. However, among 13 sites of the hydrolysis of H3 by anti-H3 IgGs and 14 sites of its hydrolysis with anti-MBP IgGs, only two sites coincide (K23-A24 and R26-K27) (Figure 4). Moreover, anti-MBP IgGs cleaves H3 histone in four sites at its C-terminal sequences, where there are no cleavage sites for anti-H3 IgGs. The N-terminal zones of H4 cleavage sites corresponding to anti-H4 and anti-MBP IgGs overlap only partially, and only three of the sites of hydrolysis of H4 by anti-MBP (9 sites) and of anti-H4 (7 sites) are the same (Figure 4). Thus, it is obvious that anti-MBP IgGs do not contain perceptible impurities of anti-H3 and anti-H4 IgGs and vice versa. The data obtained indicate that anti-MBP IgGs can bind to H3 and H4 histones and then hydrolyze these histones showing enzymatic cross-reactivity and vice versa. Consequently, antibodies against H3 histone and against MBP possess not only the ability to bind to each other but also cross-catalytic activity in the hydrolysis of these proteins. A similar situation was observed for antibodies against H4 histone and against MBP.

A feature of H3 and MBP is that it is the sequences of four MBP protein clusters that contain the MBP cleavage sites in the case of Abs against this protein are homologous to H3 protein clusters in which are located the sites of H3 cleavage by anti-histone and by anti-MBP Abs (Figure 7B,C and Figure 8B). A nearly similar situation was observed for protein clusters and sites of cleavage in the case of H4 and MBP (Figure 8D,E). Apparently, for this type of enzymatic cross-reactivity, it may be important that, in the case of MBP and histones, the homologous fragments of these proteins are their AGDs located on their surfaces, which are available for active centers of antibodies against these proteins.

Taken together, we demonstrated that IgGs against H3 and H4 and against MBP possess not only cross-complexation but also demonstrate catalytic cross-reactivity.

Previously, it was shown that MBP is hydrolyzed by antibodies against H1, H2A, and H2B histones and vice versa. In addition, MBP has several sequences showing a high level of homology similar to H3 and H4 with the protein sequences of each of the three histones: H1, H2A, and H2B [12,63]. An interesting feature of the cross-catalytic hydrolysis of H1, H2A, H2B [12,63], H3, and H4 histones that the sites of their hydrolysis by specific antibodies against each of these five histones and by IgGs against MBP practically do not coincide. As is shown above, among 13 sites of hydrolysis of H3 by IgGs against H3 and 14 sites of its splitting by anti-MBP IgGs, only two sites of the hydrolysis are the same. Between seven cleavage sites of H4 with IgGs against H4 and 9 sites of this histone hydrolysis by antibodies against MBP, only three sites were the same. In addition, anti-H1 IgGs hydrolyze H1 histone at five sites of only one cluster of the protein, while anti-MBP Abs split this histone at two clusters containing 17 sites of the cleavage; only two of all cleavage sites coincide [12]. Among fourteen sites of hydrolysis of H2A by IgGs against H2A and ten sites by anti-MBP IgGs, only one site of splitting was the same for these abzymes [63]. Eleven splitting cleavage sites of H2B with IgGs against H2B and 10 sites of its hydrolysis with antibodies against MBP were completely different [63]. Substantially, the sites of histones splitting by anti-histones and anti-MBP antibodies correspond to the antigenic determinants of the hydrolyzed histones. However, in these extended antigenic determinants sequences, anti-histone and anti-MBP IgGs hydrolyze histones in the main at different sites [12,63].

It is believed that some autoimmune pathologies may be a consequence of bacterial and viral infections [71,72]. The first immune system of humans can produce Abs against parasites or viral proteins, and then it may switch to the synthesis of auto-Abs against host antigens due to molecular mimicry between human and viral or bacterial proteins, alteration of host antigens, abnormal expression of immunoregulatory molecules, and activation of the anti-idiotypic network.

Abzymes against MBP hydrolyze the myelin basic protein of axonal envelopes of nerve tissues and therefore are extremely harmful to humans [20]. Abzymes hydrolyzing MBP were found detected in the blood sera of HIV-infected patients [12], as well as in the case of MS [26,27,28], SLE [32,33,34], and schizophrenia [35]. Interestingly, all these diseases are more or less related to the violation of the nervous system and mental disorders [71,72,73,74].

## 4. Materials and Methods

### 4.1. Materials and Chemicals

All chemicals, five histones (an equimolar mixture of H1, H2A, H2B, H3, and H4), and homogeneous human H3 and H4 were from Sigma (St. Louis, MO, USA). Protein G-Sepharose and Superdex 200 HR 10/30 column were from GE Healthcare (GE Healthcare, New York, NY, USA). Human myelin basic protein (MBP) was from the Molecular Diagnostics and Therapy Center of DBRC (Moscow, Russia). Histone- and MBP-Sepharoses were prepared by standard manufacturer’s protocol using BrCN-activated Sepharose (Sigma, St. Louis, MO, USA), MBP, the mixture of five histones as well as free H3, and H4.

Sera samples of 32 patients (18–40 yr. old; women and men) were used. In accordance with the classification of the Center for Disease Control and Prevention, 19 patients correspond to the stage of generalized lymphadenopathy (GL) and 13 humans to the stage of pre-AIDS. The blood sampling protocol was supported by Novosibirsk State Medical University Ethics Committee (number 105-HIV; 07. 2010). This Standing commission endorsed this study according to Helsinki ethics committee guidelines (permission number 72-H). All patients gave written agreement to use blood samples for scientific purposes.

High or detectable activity in the hydrolysis of H3 and H4 was revealed in the case of IgGs from 18 of 32 patients (10 with pre-AIDS and 8 patients with GL) [11]. In this study, 18 IgGs with increased activity in the hydrolysis of H3 and H4 histones were used.

### 4.2. Antibody Purification

Electrophoretically homogeneous polyclonal IgGs from blood sera of HIV-infected patients were first isolated by affinity chromatography on Protein G-Sepharose and then by the following purification by Fast protein liquid chromatography (FPLC; gel filtration) on Superdex 200 HR 10/30 column as in [11,37,38,39]. Central parts of IgGs peaks after gel filtration and filtration through filters (pore size 0.1 µm) were used for additional purification.

Removal of all IgGs against five histones from total preparation of polyclonal Abs was carried out using histone5H-Sepharose bearing immobilized five histones (H1, H2A, H2B, H3, and H4), equilibrated in buffer A (20 mM Tris-HCl, pH 7.5). After Abs loading, to get the fraction containing IgGs against five histones, the column was washed to zero optical density with buffer A. Adsorbed IgGs against five histones were eluted using buffer A containing NaCl (0.2 and 3.0 M) and finally by acidic buffer (0.1 M glycine-HCl, pH 2.6). The fraction eluted from histone5-Sepharose at loading was used for purification of anti-MBP IgGs by affinity chromatography on the column MBP-Sepharose column (5 mL) equilibrated in buffer A. After the column washing to zero optical density with buffer A, adsorbed anti-MBP IgGs were eluted using buffer A containing NaCl (0.2 and 3.0 M) and then by buffer with pH.2.6 similar to that for histone5-Sepharose. Then, the mixture of fractions of anti-MBP antibodies eluted with 3.0 M NaCl and acid buffer was again run through a column of histone5-Sepharose to remove hypothetically possible impurities of IgGs against histones. The fraction eluted upon the loading was named anti-MBP IgGs.

The mixture of IgG fractions having an affinity for histone5-Sepharose (eluted with 3 M NaCl and acidic buffer) was subjected to re-chromatography on MBP-Sepharose to remove potentially possible impurities of IgGs against MBP. The fraction eluted at loading and containing IgGs against hive histones (IgG-5histones) was used for the isolation of IgGs against H3 and H4 ones.

Then, IgG-5histones fraction was chromatographed on Sepharose containing immobilized H3 (H3-Sepharose) and H4 (H4-Sepharose) histones. Similar to the above chromatographies, IgGs without and with low affinity to the sorbents were eluted first with buffer A and then with this buffer containing 0.2 M NaCl. IgGs against H3 and H4 histones were specifically eluted with acidic buffer. These fractions were named anti-H3 IgGs and anti-H4 IgGs, respectively. SDS-PAGE (sodium dodecyl sulfate-polyacrylamide gel electrophoresis) analysis of IgGs for homogeneity was carried out in 4–17% gradient gels (0.1% SDS); the polypeptides were visualized using silver staining as in [11,12]. To exclude possible traces of canonical proteases, the IgGs against H3 and H4 histones and to MBP were subjected for SDS-PAGE with the following assay of MBP- and histones-hydrolyzing activities using eluates of gel fragments as in [11,37,38,39]. It was shown that only intact IgGs possess protease activity, and no other protein bands or catalytic activities in different fragments of gel were found.

### 4.3. ELISA of Anti-MBP and Anti-Histones Autoantibodies

Anti-MBP, as well as anti-H3 and anti-H4 histones Abs, were measured using homogeneous preparations of anti-MBP, anti-H3, and anti-H4 IgGs, according to [11,12,37,38,39,40,41]. The conditions used in this work correspond to the linear parts of the dependence of the enzyme-linked immunosorbent assay (ELISA) signal on the concentrations of antigens used (40–55% from the plateau). To ELISA strips, sodium carbonate buffer (50 μL, pH 9.6) containing 0.01 mg/mL MBP, H3, or H4 histones was added for their incubation overnight at 22 °C. All strips were first treated with buffer TBS containing 0.01% NaN_3_ and 0.05% Triton X-100 and then two times with TBS without Triton X-100. To block the surfaces of the strip, they were treated for 2 h at 37 °C using TBS supplemented with 0.2% egg albumin as well as 0.01% NaN_3_. The strips were then washed 8 times with water and then with TBS supplemented with 0.01% NaN_3_. TBS containing anti-MBP, anti-H3 or anti-H4 IgGs, 0.2% egg albumin, 0.01% NaN_3_ and 0.05% Triton X-100 (100 μL) was added to the strips for 2 h (37 °C). After washing all strips with water (9 times), 100 μL of TBS buffer containing NaN_3_ and egg albumin was added for additional incubation for 2 h at 37 °C and washed 9 times with water. Then they were incubated with TBS (100 μL) containing 1 μg/mL conjugate of anti-human monoclonal IgGs with horseradish peroxidase at 37 °C for 30 min and washed 9 times again with water. After the addition of citric-phosphate buffer (50 μL) containing H_2_O_2_ and 3,3′,5,5′-tetramethylbenzidine, all strips were incubated for 15 min at 22 °C; the reaction was stopped by adding of 50% H_2_SO_4_ (50 μL). The relative concentrations of antibodies against MBP, H3, and H4 histones in anti-MBP, anti-H3, and anti-H4 IgGs preparations were expressed as a difference in optical density (A_450_; an average of three measurements) corresponding to samples dissected with and without the above IgGs against MBP, H3, and H4.

### 4.4. Proteolytic Activity Assay

For analysis of proteolytic activity, the reaction mixtures (10–20 μL) contained 20 mM Tris-HCl (pH 7.5), 0.7–1.0 mg/mL MBP, H3 or H4 histones, and 0.01–0.05 mg/mL IgGs against MBP, H3 and H4 according to [11,12,37,38,39,40]. All mixtures were incubated at 37 °C during 1–5 h. The reaction was then stopped by the addition of the solution containing 0.1 % SDS. The efficiency of hydrolysis of MBP, H3, or H4 histones was analyzed using SDS-PAGE in 20% gel. All polypeptides were visualized using Coomassie Blue or silver staining. The gels were imaged by scanning with the following quantified by Image Quant v5.2 software (Media Cybernetics, L.P.; New York, USA). The relative activities of IgGs were evaluated from the decrease in these initial non-hydrolyzed proteins corresponding to their incubation without Abs in comparison to their content after incubation with IgGs against H3, H4, and MBP.

### 4.5. Kinetic Analysis

In all analyses, we used purified polyclonal IgGs against H3, H4, and MBP, which contain both antibodies without activity and with proteolytic activity. Therefore, it was possible to assess the apparent affinity of H3 and H4 substrates directly to abzymes only through measuring the *K*_m_ values. The initial rate data obtained using increasing concentrations of H3 and H4 were consistent with the Michaelis-Menten kinetics. The *K*_M_ and *k*_cat_ values were estimated from the dependencies of *V* versus [H3] or [H4] by non-linear least-squares fitting using Microcal Origin v5.0 software (Media Cybernetics, L.P.; USA) and presented using linear transformations of a Lineweaver–Burk plot [57]. The *k*_cat_ values (calculated from V_max,_ M/min/[IgG], M) are given as mean ± standard deviation of three independent experiments for each substrate (H3 and H4) in their hydrolysis by IgGs (0.02–0.04 mg/mL) against MBP, H3, and H4 histones. Errors in the values were within 15–20%.

### 4.6. MALDI-TOF Analysis of Histones Hydrolysis

H3 or H4 were hydrolyzed during 0–36 h using anti-MBP, anti-H3, and anti-H4 IgGs as described above. The aliquots of all reaction mixtures (1–2 µL) were analyzed over time by MALDI mass spectrometry. The analysis of products of the H3 or H4 histones hydrolysis was passed using the Reflex III system (Bruker Frankfurt, Germany): 3 ns pulse duration, 337-nm nitrogen laser VSL-337 ND. 2,5-dihydroxybenzoic or sinapinic acids were used as the two alternative matrixes. The proteins with low molecular mass (MMs) <10 kDa was analyzed using the first matrix, while the second matrix was used for the analysis of proteins and peptides with higher MMs (>10 kDa). To 1.5 µL of the matrixes and 1.5 µL of 0.2% trifluoroacetic acid, 1.5 µL of the solutions containing hydrolyzed histones H3 or H4 were added, and 1–2 µL of the final mixtures were applied on the MALDI plates that were air-dried for the analysis. Calibrations of all MALDI spectra were carried out using mixtures of oligopeptides (OPs) and proteins standards II and I (Bruker Daltonic, Berlin, Germany) in the internal and/or external calibration mode. The analysis of MMs and specific sites of H3 or H4 hydrolysis by different IgGs was performed using Protein Calculator (http://www.bioinformatics.org/sms/prot_mw.html).

### 4.7. Analysis of Sequence Homology

The homology analysis between peptides and protein sequences was carried out using *lalign* (http://www.ch.embnet.org/software/LALIGN_form.html). This program was used to analyze possible homology between the complete protein sequence of myelin basic protein with complete sequences of H3 and H4 histones.

### 4.8. Statistical Analysis

The results correspond to the average values (mean ± standard deviation) from 3 independent experiments.

## 5. Conclusions

We have demonstrated that IgGs against H3 and H4 histones and against MBP possess not only cross-complexation, but also manifest catalytic cross-reactivity. It cannot be excluded that the catalytic cross-reactivity of IgGs hydrolyzing H1, H3, and H4 histones and MBP, which are able to hydrolyze MBP of the myelin sheaths of nerve tissues, can have a momentous role in the development of neurodegenerative and neuropsychiatric changes in the case of all these diseases.

## Figures and Tables

**Table 1:** The *K_m_* and *k_cat_* values for H3 and H4 histones**.**

Substrate	Abs	*K*_M_, µM	*k*_cat_, min^−1^
H3	anti-MBP IgGs	90.0 ± 8.0	0.02 ± 0.002
H4	anti-MBP IgGs	143.0 ± 10.0	0.037 ± 0.004
H3	anti-H3 IgGs	76.9 ± 8.0	0.09 ± 0.008
H4	anti-H4 IgGs	113.0 ± 12.0	0.076 ± 0.008

## Data Availability

All data are given in the article and Attached files.

## Supplementary Materials

The following are available online, Figure S1: Homology of complete sequences of myelin basic protein and H3 histone.

## Sample Availability

Samples of antibodies are available from the authors.

## Abbreviations

Ab	antibody
Abz	abzyme
AI	autoimmune
AA	amino acid
AIDS	acquired immune deficiency syndrome
GL	generalized lymphadenopathy
AGDs	antigenic determinants
HIV-1	human immunodeficiency virus type 1
OP	oligopeptide
MALDI-TOF	matrix-assisted laser ionization/desorption time-of-flight mass spectrometry
MS	multiple sclerosis
MBP	myelin basic protein
SDS-PAGE	sodium dodecyl sulfate-polyacrylamide gel electrophoresis
SLE	systemic lupus erythematosus
