# Supplementary material for: HIV-Infected Patients: Cross Site-Specific Hydrolysis of H3 and H4 Histones and Myelin Basic Protein with Antibodies against These Three Proteins
Source: Molecules. 2021 Jan 9;26(2):316. doi: 10.3390/molecules26020316 (PMC7826842; doi:10.3390/molecules26020316)
Supplement: Supplementary file 1 [file molecules-26-00316-s001.pdf]

Supplementary Figure S1

Homology of complete sequences of myelin basic protein and H3 histone

```
 Waterman-Eggert score: 118;  11.7 bits; E(1) <  1
 26.4% identity (47.9% similar) in 163 aa overlap (30-170:2-129)

      30        40        50         60        70          80
MBP    RHRDTGILDSLGRFFGSDRGAPKRGSGKD-GHHAARTTHYGSLPQKAQG--HRPQDENP-
       : ..:.   : :   :  . :: :   :. . .:::  .  : :  : :  ..:.   :
H3     RTKQTA-RKSTG---G--K-AP-R---KQLATKAAR--K--SAP--ATGGVKKPHRYRPG
               10                  20            30          40

          90       100       110            120       130
MBP    VVHFFKNIVTPRTPPPSQGKGRGLSLSR---FSWGA-E-GQKPGFGYGGRASD--YKS-A
       .: . ..:   :     : :.  : : :   :.  . : .:   :    . .:  ..: :
H3     TVAL-REI---RR---YQ-KSTEL-LIRKLPFQRLVREIAQ--DF----K-TDLRFQSSA
            50               60         70               80

       140        150           160            170
MBP    HKGLKGHDA-QGTLSKIFK---LGG-RDSR-SGSP----MARR
         .:.  .: .. :  .:.   : . . .: .  :    .:::
H3     VMALQ--EACEAYLVGLFEDTNLCAIHAKRVTIMPKDIQLARR
       90         100       110       120
```

Homology of complete sequences of myelin basic protein and H4 histone

```
>>H4 102 bp                                                  (102 aa)
 Waterman-Eggert score: 107;  13.9 bits; E(1) <  0.68
30.0% identity (45.0% similar) in 140 aa overlap (39-158:1-102)

       40        50        60        70        80        90
MBP    SLGRFFGSDRGAPKRGSGKDGHHAARTTHYGSLPQKAQGHRPQDENPVVHFFKNIVTPRT
       : ::  :  .:. : : :: :  : :  :     .:.   :  : : .    ..: : .
H4     S-GR--G--KGG-K-GLGKGG--AKR--H-----RKVL--R--D-N-I----QGI-T-K-
                      10                 20                   30

      100        110            120          130           140
MBP    PPPSQ-GKGRGLSLSRFSWG---AE--G-QKPGFGYGG--R-ASDYKSAH---KGLKGHD
       :   . .. :: ...:.: :    :  :  :  :   .  : :  : . :   : . . :
H4     PAIRRLAR-RG-GVKRIS-GLIYEETRGVLKV-F-LENVIRDAVTY-TEHAKRKTVTAMD
               40          50        60          70         80

                150
MBP    ---A---QG-TLSKIFKLGG
```

Homology of complete sequences of histones and myelin basic protein was estimated using:

http://www.ch.embnet.org/software/LALIGN_form.html).
